# Clinical characteristics of Netherton syndrome and exploration of targeted biologic therapy: two case reports

**DOI:** 10.3389/falgy.2025.1667357

**Published:** 2025-09-03

**Authors:** Wanyan Xiang, Chengxiang Lian, Jiarong Lu, Wenjun Zheng, Qiuju Li

**Affiliations:** Department of Dermatology, First Affiliated Hospital of Guangxi Medical University, Nanning, China

**Keywords:** Netherton syndrome, biologic, dupilumab, secukinumab, treatment

## Abstract

**Background:**

Netherton syndrome (NS) is a rare, autosomal recessive disease resulting from a mutation in the pathogenic variants in the Kazal type 5 (SPINK5) gene. In recent years, the targeted treatment of biological agents has increasingly emerged as a focal point of research.

**Case reports:**

We reported a 4-month-old child and 19-year-old female, both presenting with symptoms including erythema, scaling, and recurring episodes. Subsequently, genetic testing identified a defective SPINK5 gene, leading to a diagnosis of NS. The child received treatment with dupilumab, while the 19-year-old woman alternated between using dupilumab and secukinumab. Both patients had swift and enduring enhancement of skin lesions during the follow-up period.

**Conclusion:**

NS is an uncommon and frequently misdiagnosed hereditary dermatological disease. The management strategies for this condition are diverse, and no consensus exists. We implemented various biologic regimens for distinct patients, all demonstrating favorable outcomes and satisfactory tolerance. Besides, monitoring and evaluating the long-term safety of biologics in combination is essential.

## Introduction

Netherton syndrome (NS) is a rare autosomal recessive hereditary disease caused by the mutation of the SPINK5 gene, which encodes the Serpin LEKTI (lymphoepithelial Kazal-related inhibitor) (1). LEKTI deficiency in the epidermis can result in unregulated protein hydrolysis, compromised skin barrier function, and heightened production of T helper 2 cells (Th2) cytokines (2, 3). The characteristic clinical manifestations of NS primarily include ichthyosis linearis circumflexa (ILC), overlapping brittle hair, and a distinct constitution—such as atopic dermatitis (AD), elevated serum immunoglobulin E (IgE), and increased eosinophil count in routine blood tests. These features, along with asthma, allergic cough, allergic conjunctivitis, allergic rhinitis, pruritus, and urticaria, often lead to misdiagnosis as psoriasis or atopic dermatitis (4).

Presently, there is no specific and effective treatment for NS. Current topical therapies include topical moisturizers, glucocorticoids, calcineurin inhibitors, or antibacterial agents (5). In recent years, the targeted treatment of biological agents has increasingly emerged as a research hotspot. Recent case studies indicate that treatment with IL-17A or IL-4R*α* antagonists has yielded partial improvement in NS patients; nonetheless, some individuals continue to exhibit inadequate management with monotherapy (6). This report details two NS cases well managed with single-use and combination biological treatments.

## Case presentation

### Case 1

A 4-month-old infant exhibited persistent systemic erythema and scaling for over 4 months. Upon birth, the infant's entire body exhibited erythema, followed by the appearance of erythematous papules and scales after 2 days.

The infant first sought medical attention at external hospitals, where he was misdiagnosed with AD or psoriasis. Although the disease initially improved with extensive symptomatic treatment (including topical mild corticosteroids, oral antihistamines, and emollients), the rash recurred frequently, accompanied by significant pruritus.

**Figure 1:**
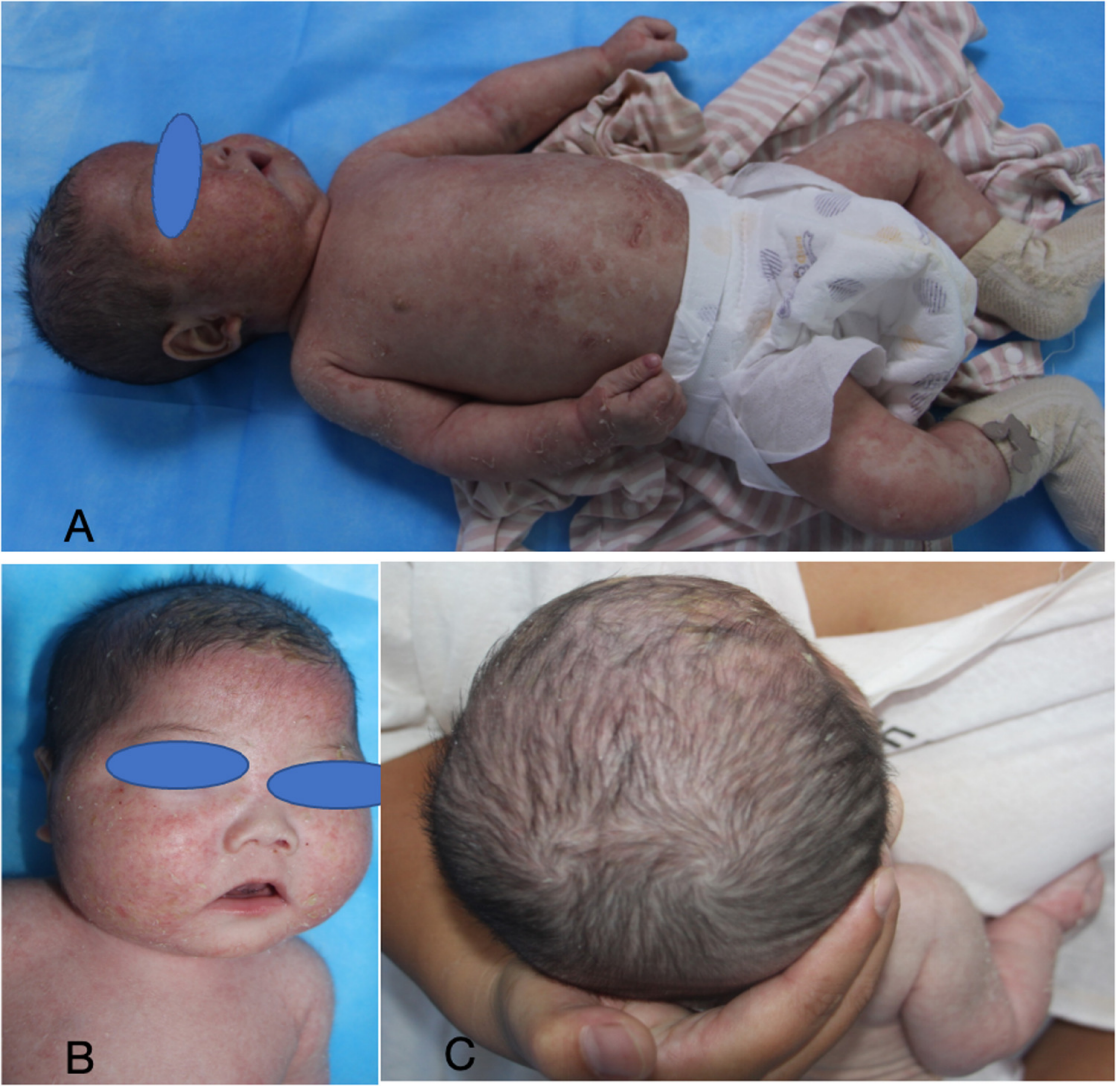
The eyebrows exhibit sparsity, the capillaries on both cheeks are engorged, and the head, face, trunk, and limbs display erythema, partially in a ring or map formation, accompanied by thin scales on the surface, with localized scabs and scratches evident.

**Figure 2:**
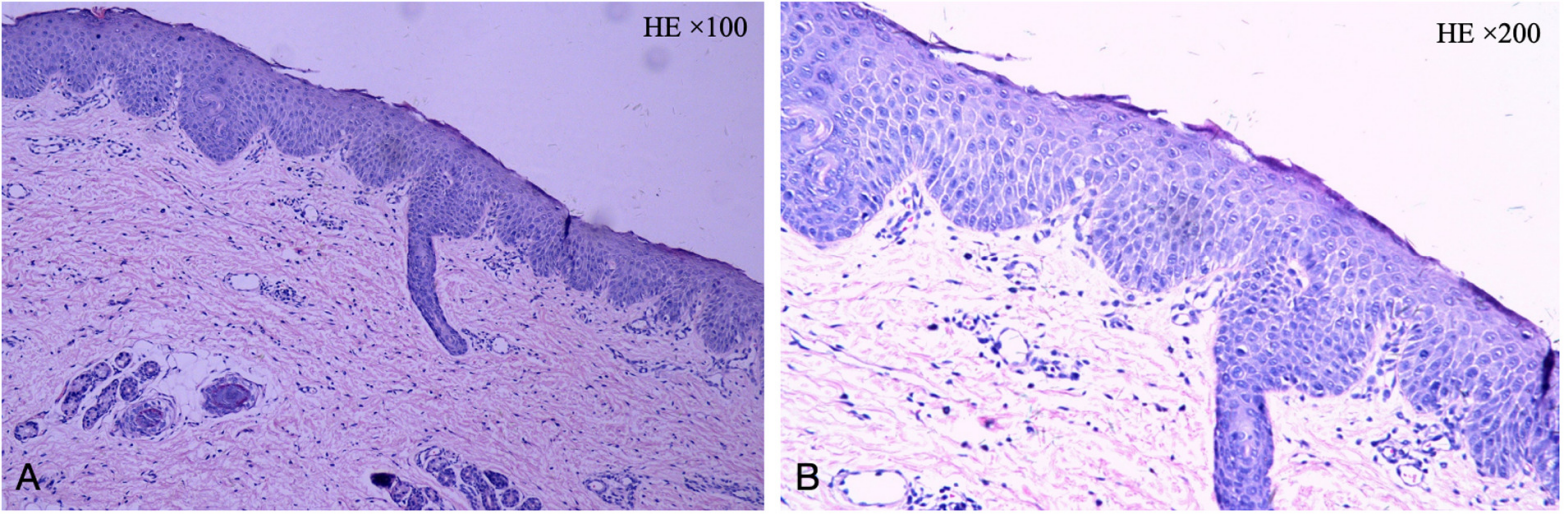
The pathology of the skin lesion on the left upper arm: excessive keratinization, attenuation of the granular layer, mild psoriasis-like hyperplasia in the spinous layer, and little perivascular inflammation in the superficial dermis. (**A**: HE × 100, **B**: HE × 200).

Subsequently, the patient was referred to our hospital for further evaluation. After arriving at our hospital, a detailed physical examination showed that his eyebrows were sparse and the capillaries on his cheeks were engorged. He has marked erythema on his head, face, trunk, and limbs, characterized by a circular or map-like pattern with thin scales and local scabs and scratches on the surface (Figure 1). Blood results indicated a serum IgE level of 36.9 IU/ml. The pathology of the skin lesion on the left upper arm revealed excessive keratinization, attenuation of the granular layer, mild psoriasis-like hyperplasia in the spinous layer, and little perivascular inflammation in the superficial dermis (Figure 2). Due to the absence of response to standard treatment and the onset of symptoms at birth, genetic testing was performed, which validated an SPINK5 mutation [2 heterozygous mutations: c.2423C>T (p.T808I) and c.2468delA (p.K823Rfs*101)].

**Figure 3:**
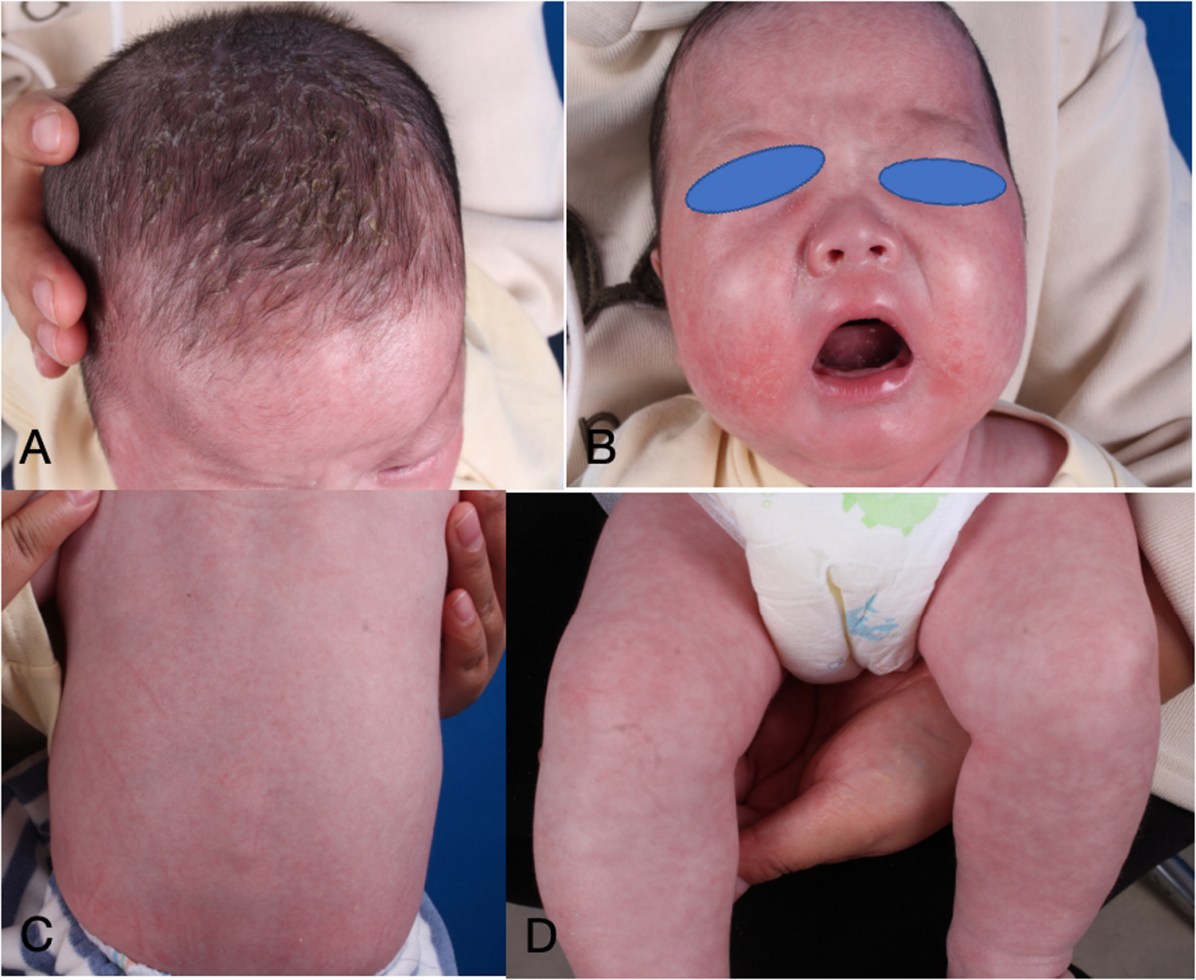
**(A–D)** Dermatological and trichological manifestations following 2 months of dupilumab treatment.

**Figure 4:**
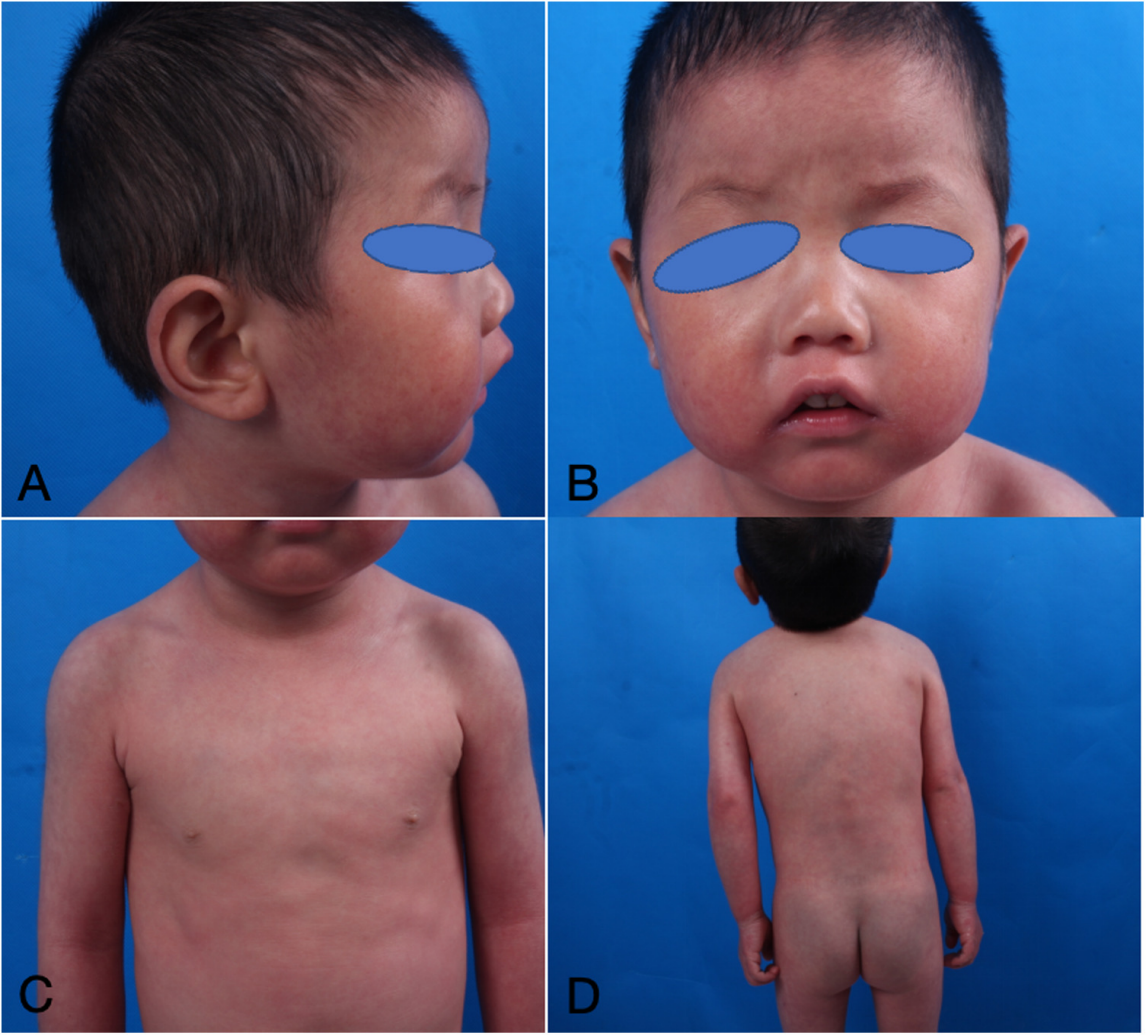
**(A**–**D)** Dermatological and trichological manifestations of dupilumab treatment for more than 2 years.

The infant commenced subcutaneous dupilumab at a dosage of 200 mg once monthly. Following 2 months of dupilumab treatment, the skin flushing and ichthyic symptoms ameliorated (Figure 3), and the scoring atopic dermatitis index (SCORAD) decreased from 77.17 at baseline to 45.32 after 2 months of dupilumab treatment. The effect persisted until he reached 3 years old (Figure 4).

### Case 2

**Figure 5:**
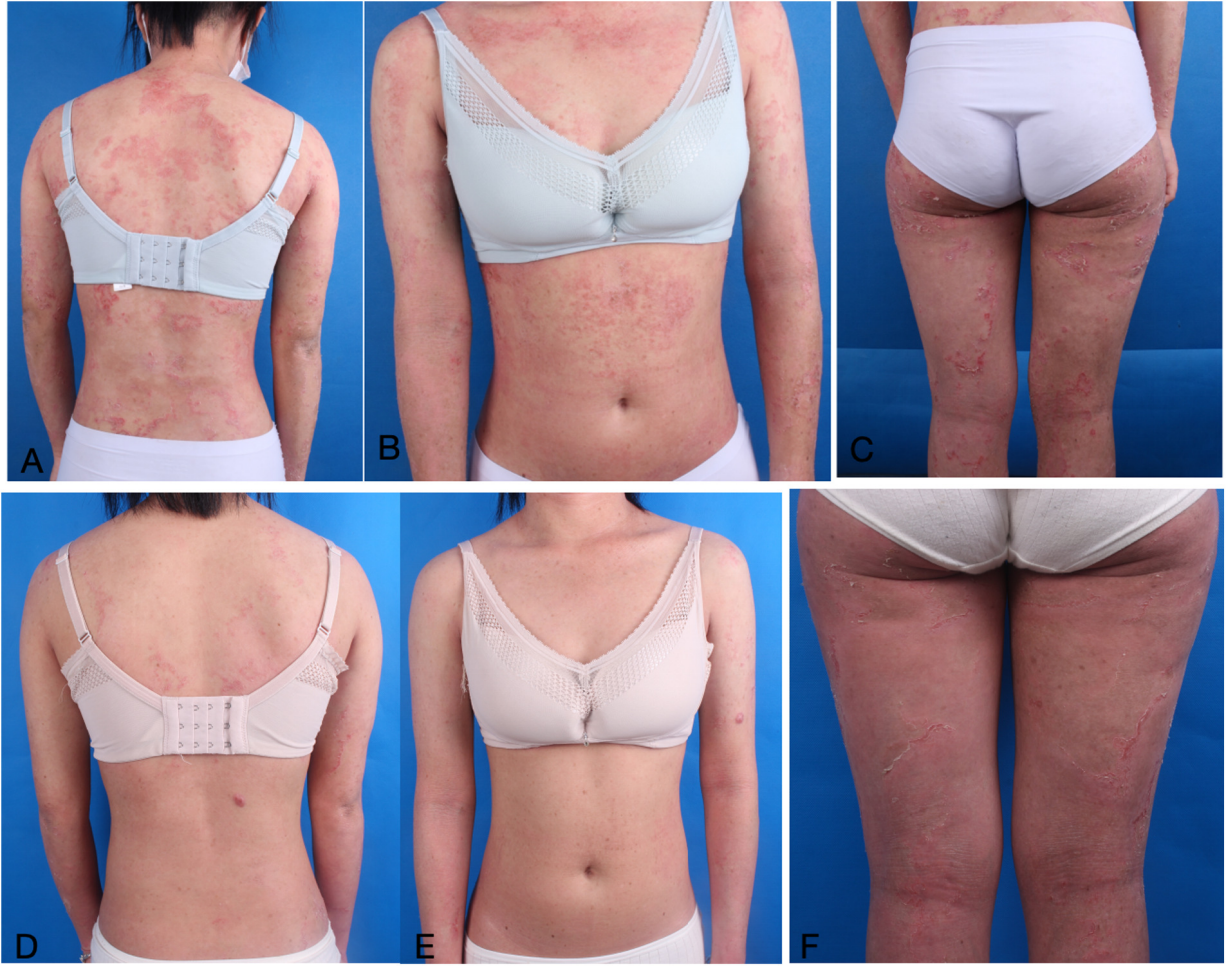
**(A**–**C)** Illustrates intussusception brittle hair and congenital circular linear ichthyosis. **(D**–**F)** Dermatological and trichologic manifestations after dupilumab combined with secukinumab.

**Figure 6:**
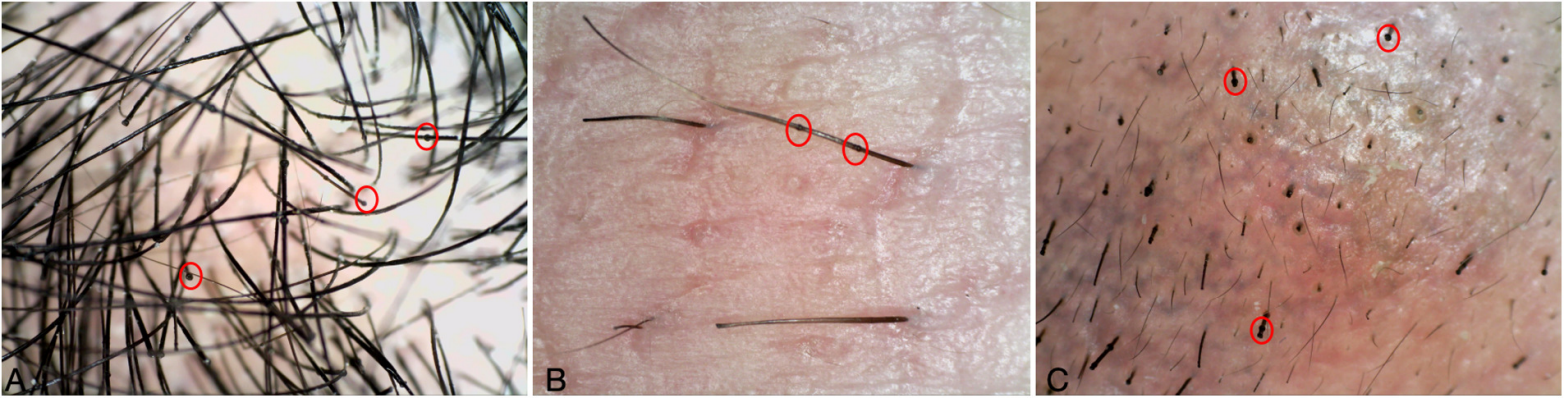
**(A**–**C)** Dermatoscopy of hair, eyebrows, and axillary hair: bamboo knot hair, golf tee hair and matchstick hair.

A 19-year-old woman went to the dermatology clinic with over 15 years of serrated erythema and serpiginous desquamation at the edges of a ring (Figures 5A–C). The dermatological examination conducted at our hospital revealed severe hair breakage, sparse eyebrows, and underarm hair loss, with dermoscopy showing “bamboo knot,” “golf tee,” and “matchstick” hair (Figure 6). Her diagnostic journey began in childhood, when she sought treatment at multiple external hospitals. Over the years, she was repeatedly diagnosed with AD or psoriasis based on clinical appearance and received various treatments, including topical steroids, antihistamines, cyclosporine, secukinumab monotherapy, and JAK inhibitors (abrocitinib and upadacitinib). However, her symptoms relapsed shortly after temporary improvement, prompting persistent dissatisfaction with prior management.

The medical history indicated that she had been unwell since childhood and exhibited small stature, measuring 145 cm in height. Blood tests at our hospital showed an eosinophil percentage (EO%) of 5.00%, an eosinophil count of 0.28 × 10^9^/L, and a serum IgE level of 1,804.2 IU/ml.

**Figure 7:**
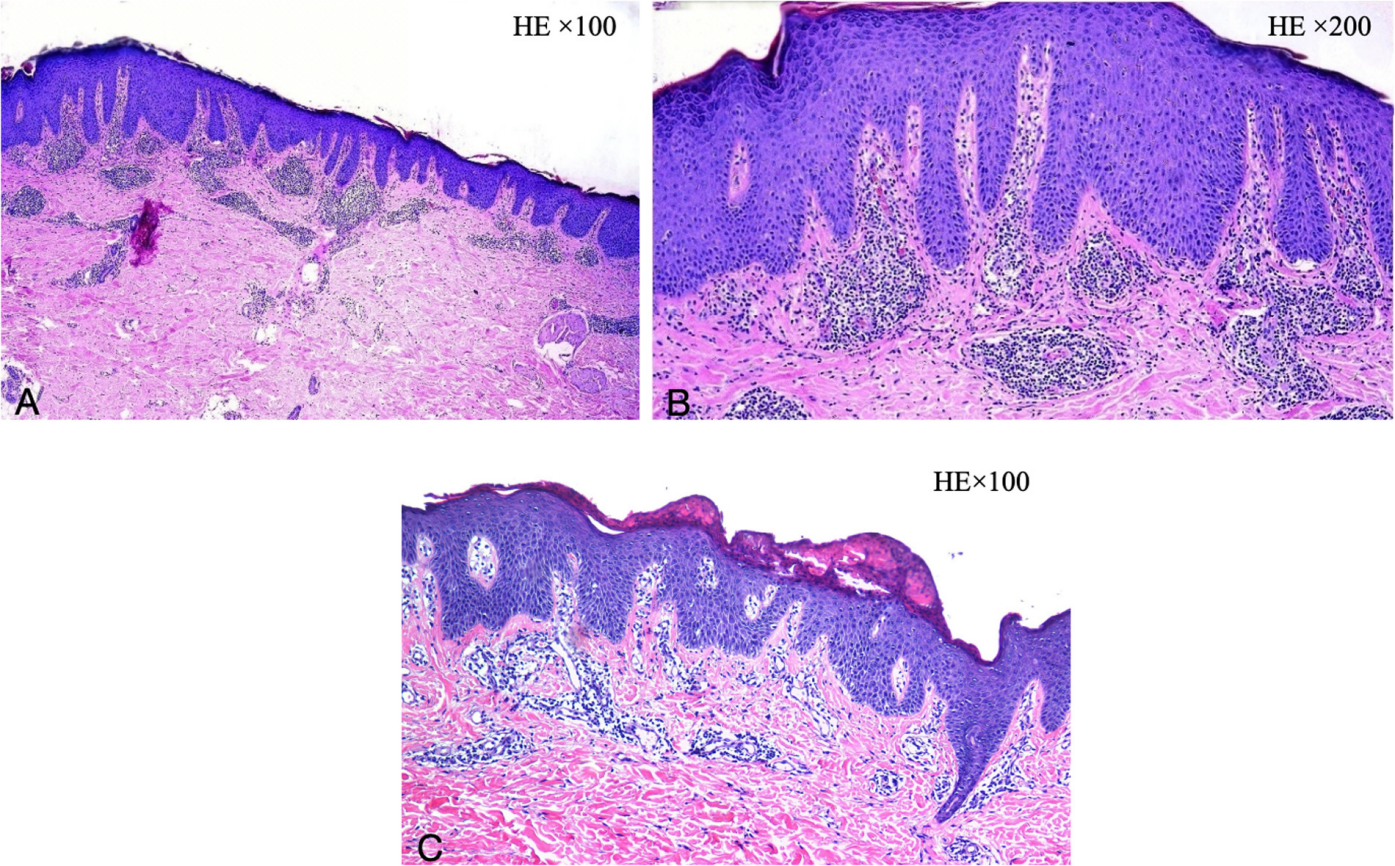
**(A,B)** Initial dermatological pathology: hypokeratotic keratosis in the stratum corneum, psoriasis of the epidermis, and mild perivascular inflammation in the superficial dermis. **(C)** Secondary dermatological pathology: spongiform dermatitis. (**A,C**: HE × 100, **B**: HE × 200).

During her initial evaluation at our hospital, two skin biopsies were performed (Figures 7A–C): Initial pathology: hypokeratotic keratosis in the stratum corneum, epidermal psoriasis-like changes, and mild perivascular inflammation in the superficial dermis. Secondary pathology: spongiform dermatitis. Given the lifelong onset of erythema and the discrepancy between pathological findings and clinical course, we proceeded with genetic testing, which proved a mutation in the SPINK5 gene (deletion of mutation in the 5q32 region of the genome). The patient received a diagnosis of NS.

Consequently, she received a combination treatment of secukinumab 150 mg weekly and dupilumab 300 mg biweekly. After one month, the skin lesions exhibited substantial improvement. Combination therapy led to a SCORAD reduction from 73.2 to 48.1 over six months, while the Dermatology Life Quality Index (DLQI) improved from 13 to 9, reflecting the clinical enhancement observed. And no extensive recurrence was noted throughout the 6-month follow-up (Figures 5D–F).

## Discussion

NS, or SPINK5-epidermal differentiation disorders syndrome (SPINK5-sEDD), is defined by profound skin barrier impairments and persistent cutaneous inflammation associated with interleukin (IL)-36, IL-23/T helper (Th) 17 and type 2 immunological signals (7).

The diagnosis of NS may be challenging for several reasons: ILC is not always present; hair analyzed via optical microscope may show no abnormalities in hair stems, as only 20%–50% of hair is impacted; and atopic symptoms may obscure the clinical presentation (8). The predominant histological features of NS are psoriasis-like hyperplasia, along with dermal inflammation atrophy, and diminishment of the stratum granulosum. Histology lacks specificity; nonetheless, a negative LEKTI immunostaining may indicate the diagnosis. Several cases are LEKTI positive, but genetic testing is essential for diagnosis confirmation (1, 9).

Currently, there is no definitive treatment method for NS patients, primarily consisting of symptomatic supportive care, which includes topical emollients, glucocorticoids, calcipotriol, narrowband UV-B phototherapy, psoralen–UV-A phototherapy and retinoid-like phototherapy, exhibiting variable efficacy (3). However, if the patient's skin barrier function is compromised, it is essential to consider the possibility of transdermal medication absorption (2).

In response to the pathogenesis of NS, novel pharmacological agents were formulated to mitigate the hyperactivity of the upper epidermal kinin kinase protein through either topical application or systemic delivery of kinin kinase inhibitors. Currently, numerous researchers employ biological treatments such as infliximab (10), omalizumab (11), secukinumab (12), and dupilumab, together with JAK inhibitors (13, 14) to alleviate skin inflammation. In our case 1, we noted considerable enhancement in this infant's cutaneous lesions and hair manifestations following 2 months of exclusive dupilumab treatment, and the effect lasted until 3 years of age.

Recent case studies indicated that while treatment with IL-17A or IL-4R antagonists is well tolerated, it yields only partial improvement in some patients with NS (6, 15, 16). This suggests that monotherapy may be insufficient to manage mixed inflammation in this type of NS. As in our second case, erythema and scales were obvious from childhood. The patient exhibited thinning and scant hair, along with elevated IgE levels. A dermatoscopic examination of the hair reveals overlapping weak strands. Genetic analysis confirmed mutations in the SPINK5 gene, corroborating the diagnosis of NS. The patient sequentially utilized secukinumab and abrocitinib; however, the skin lesions exhibited only transient improvement. We opted for combination therapy of secukinumab and dupilumab. And surprisingly, there was a marked enhancement in skin lesions and hair symptoms, with a sustained impact noted after 6 months of treatment.

Notably, we used AD-specific indicators for quantitative assessment, and the results showed that after treatment, SCORAD decreased significantly in both patients, and DLQI of the second patient was significantly improved, which also confirmed that the appropriate biologics could improve the signs, symptoms, and quality of life of NS patients.

A proof-of-concept trial showed swift and significant improvements in pruritus and cutaneous lesions, including hair, as early as 4 weeks following combination therapy with secukinumab and dupilumab for children with NS (17). It resembles our observations in the second patient. Of course, further research to verify the effectiveness and long-term safety of the combination of biologics for NS is also crucial.

## Conclusion

NS poses diagnostic challenges due to its variable presentation and overlapping features with common dermatoses like AD or psoriasis, often leading to prolonged misdiagnosis. These cases highlight the critical role of early recognition and genetic testing for definitive diagnosis, and demonstrate that combination therapy with secukinumab and dupilumab effectively targets the mixed inflammatory pathways in NS, significantly improving both clinical symptoms and quality of life metrics (SCORAD and DLQI) with sustained efficacy. These findings support the potential of combined biologic agents as a promising therapeutic strategy for NS, emphasizing the need for further research on long-term safety and efficacy.

## Data Availability

The raw data supporting the conclusions of this article will be made available by the authors, without undue reservation.
