# Supplementary material for: Clinical characteristics of Netherton syndrome and exploration of targeted biologic therapy: two case reports
Source: Front Allergy. 2025 Sep 3;6:1667357. doi: 10.3389/falgy.2025.1667357 (PMC12441801; doi:10.3389/falgy.2025.1667357)
Supplement: Supplementary file 1 [file Datasheet1.pdf]

CARE case report guidelines

CARE Checklist of information to include when writing a case report

(a)EY-NC-N

pic	Item	Checklist item description	Reported on Line
**Title**	1	The diagnosis or intervention of primary focus followed by the words "case report"…… ……….……	YES
**Key Words**	2	2to 5 key words that identify diagnoses or interventions in this case report,including "case report"..	YES
**Abstract (no references)**	3a	Introduction:What is unique about this case and what does it add to the scientific iterature?……………	YES
	3b	Main symptoms and/or important dlinical finding.	YES
	3c	The main diagnoses,therapeutic interventions,and outcomes...… ………… ……… ……………………..	YES
	3d	Conclusion—What is the main "take-away"lesson(s)from this case?..………………………………	YES
**Introduction**	4	One or two paragraphs summarizing why this case is unique**(may include references)** ....……	YES
**Patient Information**	5a	De-identified patient specific information. ....……………………	YES
	5b	Primary concerns and symptoms of the patient..…… …… …… …… …… ………	YES
	5c	Medical,family,and psycho-social history including relevant genetic information.................	YES
	5d	Relevant past interventions with outcomes ..………………………………………………	YES
Clinical Findings	6	Describe significant physical examination(PE)and important clinical finding..………………	YES
Timeline	7	Historical and current information from this episode of care organized as a timeline...............	YES
Diagnostic Assessment	8a	Diagnostic testing(such as PE,laboratory testing,imaging,surveys) ..………………	YES
	8b	Diagnostic challenges (such as access to testing,financial,or cultural)………………	YES
	8c	Diagnosis(including other diagnoses considered) ……… .... … ……… … …..	YES
	8d	Prognosis (such as staging in oncology)where applicable...…………… …………………..	YES
**Therapeutic Intervention**	9a	Types of therapeutic intervention(such as phamacologic,surgical,preventive,self-care)....……………...	YES
	9b	Administration of therapeutic intervention (such as dosage,strength,duration) ..………………………....	YES
	9c	Changes in therapeutic intervention(with rationale) ...………	YES
**Follow-up and Outcomes**	10a	Cinician and patient-assessed outcomes (if available)...………………………………………	YES
	10b	Important follow-up diagnostic and other test results ..… ……… ……… ………………………	YES
	10c	Intervention adherence and tolerability(How was this assessed?).…… .... …………………..	YES
	10d	Adverse and unanticipated events ...…………………………………………………………...	YES
**Discussion**	11a	A scientific discussion of the strengths AND limitations associated with this case report..…………… ……	YES
	11b	Discussion of the relevant medical literature **with references** ...…………… …….............	YES
	11c	The scientificrationale for any conclusions (including assessment of possible causes)…… .... …	YES
	11d	The primary "take-away"lessons of this case report (without references)in a one paragraph condusion.......	YES
**Patient Perspective**	12	The patient should share their perspective in one to two paragraphs on the treatment(s)they received.......	YES
**Informed Consent**	13	Did the patient give informed consent?Please provide if requested…………………	**Yes** ☑ **No** ☐
